# Molecular Basis for Dysregulated Activation of NKX2‐5 in the Vascular Remodeling of Systemic Sclerosis

**DOI:** 10.1002/art.40419

**Published:** 2018-04-24

**Authors:** Athina Dritsoula, Ioannis Papaioannou, Sandra G. Guerra, Carmen Fonseca, Javier Martin, Ariane L. Herrick, David J. Abraham, Christopher P. Denton, Markella Ponticos

**Affiliations:** ^1^ University College London London UK; ^2^ Instituto de Parasitología y Biomédicina López‐Neyra Granada Spain; ^3^ University of Manchester Salford Royal NHS Foundation Trust and Central Manchester NHS Foundation Trust Manchester Academic Health Science Centre Manchester UK

## Abstract

**Objective:**

NKX2‐5 is a homeobox transcription factor that is required for the formation of the heart and vessels during development, with significant postnatal down‐regulation and reactivation in disease states, characterized by vascular remodeling. The purpose of this study was to investigate mechanisms that activate NKX2‐5 expression in diseased vessels, such as systemic sclerosis (scleroderma; SSc)–associated pulmonary hypertension (PH), and to identify genetic variability that potentially underlies susceptibility to specific vascular complications.

**Methods:**

We explored NKX2‐5 expression in biopsy samples from patients with SSc‐associated PH and in pulmonary artery smooth muscle cells (PASMCs) from patients with scleroderma. Disease‐associated putative functional single‐nucleotide polymorphisms (SNPs) at the *NKX2-5* locus were cloned and studied in reporter gene assays. SNP function was further examined through protein–DNA binding assays, chromatin immunoprecipitation assays, and RNA silencing analyses.

**Results:**

Increased NKX2‐5 expression in biopsy samples from patients with SSc‐associated PH was localized to remodeled vessels and PASMCs. Meta‐analysis of 2 independent scleroderma cohorts revealed an association of rs3131917 with scleroderma (*P* = 0.029). We demonstrated that disease‐associated SNPs are located in a novel functional enhancer, which increases *NKX2-5* transcriptional activity through the binding of GATA‐6, c‐Jun, and myocyte‐specific enhancer factor 2C. We also characterized an activator/coactivator transcription‐enhancer factor domain 1 (TEAD1)/Yes‐associated protein 1 (YAP1) complex, which was bound at rs3095870, another functional SNP, with TEAD1 binding the risk allele and activating the transcription of *NKX2-5*.

**Conclusion:**

*NKX2-5* is genetically associated with scleroderma, pulmonary hypertension, and fibrosis. Functional evidence revealed a regulatory mechanism that results in *NKX2-5* transcriptional activation in PASMCs through the interaction of an upstream promoter and a novel downstream enhancer. This mechanism can act as a model for NKX2‐5 activation in cardiovascular disease characterized by vascular remodeling.

NKX2‐5 is a transcription factor that belongs to the family of NK2‐homeobox DNA binding transcription activators. One of the earliest known markers of cardiac development in vertebrates 1, 2, NKX2‐5 is crucial for blood vessel development during embryogenesis 3, 4. In humans, NKX2‐5 is not expressed in normal vasculature postnatally. However, we have accumulating evidence that NKX2‐5 drives phenotypic dedifferentiation of vascular smooth muscle cells (VSMCs) in blood vessels undergoing vascular remodeling 5, 6. Vascular remodeling is the term used to describe the structural rearrangement of the vessel wall in response to inflammation, repair, or other stimuli 7. It is the hallmark of many vascular diseases, including atherosclerosis, pulmonary arterial hypertension (PAH), and scleroderma (SSc)–associated pulmonary hypertension (PH).

SSc is a multisystem disease characterized by increased dysregulation of the immune system, inflammation, extensive fibrosis of the skin and internal organs, and prominent vasculopathy 8. As in other rare heterogeneous diseases, it is likely that a combination of both genetic and environmental factors interact to cause SSc 9. The unmet need in the medical management of SSc is high, including the high rates of death from cardiorespiratory complications. SSc‐associated PH is a leading cause of death among SSc patients 8, and PH develops in 18–24% of SSc cases 10.

SSc‐associated PH occurs through several mechanisms, including World Health Organization (WHO) group I PAH, as well as WHO group II (postcapillary) and group III (lung fibrosis associated) forms. Collectively, these are termed SSc‐associated PH. PH can develop throughout the course of the disease, and recent studies suggest that 1–2% of SSc patients develop PH each year in a screened population 10, suggesting that SSc confers substantial susceptibility. All forms of PH are associated with vascular remodeling, and it is likely that overlapping molecular pathways are involved in the development of this, and possibly other, SSc‐associated vascular manifestations.

Transcriptional regulation of the murine *Nkx2-5* gene has proven to be very complex, with a number of *cis*‐ and *trans*‐acting elements over a large 23‐kb genomic region regulating expression in a temporospatial‐specific manner 11. BMP signaling is necessary for *Nkx2-5* activation through the binding of Smad and GATA transcription factors at an upstream enhancer 12, 13. Other signaling pathways, epigenetic modifications, and autoregulatory mechanisms also govern *Nkx2-5* regulation 14, 15. Despite our knowledge of the structure of the murine *Nkx2-5* gene and the high homology (87%) between the mouse and human genes, little is known about the regulation of human *NKX2-5*. Most studies focus on *NKX2-5* genetic variations, with 56 mutations and 250 single‐nucleotide polymorphisms (SNPs) having been identified, many of which are associated with types of congenital heart disease 16, 17.

Postnatal activation of developmental regulatory pathways may explain the molecular pathology of the adult disease, and genetic association of functional polymorphisms can explain the susceptibility and phenotypic variability. In this study, we investigated the regulatory mechanisms of human *NKX2-5* resulting in increased expression in adult vasculature. We delineated a transcriptional mechanism through which *NKX2-5* is activated. We also identified and validated a downstream enhancer, which results in enhanced transcriptional activation in human pulmonary artery smooth muscle cells (PASMCs).

## Patients and methods

**Patient cohorts.** A genetic study was conducted in 2 independent cohorts of Caucasian origin: UK and Spanish SSc patients. The UK cohort consisted of 1,334 SSc patients presenting to the Centre for Rheumatology at the Royal Free Hospital and the Centre for Musculoskeletal Research at the University of Manchester, as well as 901 control DNA samples matched to the patients for age, sex, and ethnicity. The Spanish cohort consisted of 1,736 SSc patients and 1,753 control DNA samples collected at the Institute of Parasitology and Biomedicine Lopez‐Neyra in Granada, Spain. The study was approved by the local ethics committees, in compliance with the Helsinki Declaration, and all participants gave written informed consent to participate in the study.

All patients had a definite diagnosis of SSc, and the majority fulfilled the American College of Rheumatology/European League Against Rheumatism 2013 criteria for the classification of SSc 18. Patients were categorized according to disease subsets (limited/diffuse) or the presence of the following autoantibodies: anticentromere, antitopoisomerase, and anti–RNA polymerase III. The presence of pulmonary fibrosis was assessed by high‐resolution computed tomography and a restrictive pattern on lung function testing. Patients with estimated systolic pulmonary arterial pressure (PAP) of ≥45 mm Hg on echocardiogram or a mean PAP of ≥25 mm Hg at the time of right‐sided heart catheterization were classified as having PH. Those without clinically significant lung fibrosis but with a confirmed pulmonary arterial wedge pressure of ≤15 mm Hg at right‐sided heart catheterization were defined as having WHO group I PAH 19. Renal crisis was diagnosed according to the acute onset of severe hypertension and acute kidney injury 20. Medical management of the study cohorts was consistent with current practice, with regular review and annual assessment for new organ‐based complications.

Extended protocols and information regarding the experimental procedures are provided in the Supplementary Methods (available on the *Arthritis & Rheumatology* web site at http://onlinelibrary.wiley.com/doi/10.1002/art.40419/abstract).

**Selection of tagging SNPs.** Tagging SNPs around the *NKX2-5* genomic region (chromosome 5: 173,272,104–173,240,312; GenBank GRCh38.p7 assembly) were selected with Tagger software 21 using SNP genotype data from the northern European population (Utah Residents [CEPH] of North and Western European ancestry [CEU]) available from the International HapMap Project, Release 28 22. All SNPs had a minor allele frequency of >0.01 and were in Hardy‐Weinberg equilibrium (cutoff value 0.001).

In silico analyses were performed using annotations from the following open‐access curated databases: Encyclopedia of DNA Elements (ENCODE) 23, TRANSFAC 24, JASPAR 25, and HaploReg 26.

**DNA extraction and genotyping.** DNA was extracted from whole blood as described elsewhere 27. SNPs rs703752, rs3131917, rs3132139, rs12514371, and rs2277923 were genotyped using TaqMan SNP genotyping assays. For rs3095870, a high‐resolution melting analysis was performed using a Type‐it high‐resolution melting polymerase chain reaction (PCR) kit (Qiagen).

**Genetic association study.** The case–control association analysis and the subphenotype analysis were performed in Plink 28. Successful call rates per SNP and per individual (≥90%) were applied. Permutation analysis was used to correct for multiple comparisons. Haplotype analysis was performed in Haploview 29.

**Cell culture, cloning, and luciferase assays.** Primary human PASMCs (catalog no. C‐12521; PromoCell) and immortalized human PASMCs (catalog no. T0558; ABM Good) were used for the in vitro experiments. *NKX2-5* gene expression in patients with SSc‐associated PH and in matched controls was assessed in primary PASMCs isolated from human tissue as described previously 30. All SMCs were cultured in media supplemented with 5% serum at 70–80% confluency. Primary cells were used between passages 3 and 9. Human PASMCs were immortalized via a lentiviral vector containing SV40.

Disease‐associated SNPs were cloned into reporter vectors and transfected into primary human PASMCs. Luciferase assays were then performed.

**RNA silencing, binding assays, and Western blotting.** Immortalized human PASMCs were transfected for 48–72 hours with small interfering RNA (siRNA) oligonucleotides specific for transcription‐enhancer factor domain 1 (TEAD1), TEAD3, and Yes‐associated protein 1 (YAP1) (On‐Target Plus SMARTpool siRNA; Dharmacon). RNA and protein were extracted and subjected to quantitative PCR (qPCR), sodium dodecyl sulfate–polyacrylamide gel electrophoresis (SDS‐PAGE), and Western blotting.

Electrophoretic mobility shift assay (EMSA), DNA–protein pull‐down assays, and chromatin immunoprecipitation (ChIP) assays were performed to identify protein complexes that bind the associated SNPs.

Nuclear (NE‐PER kit; Pierce) and total cell (radioimmunoprecipitation assay) protein extracts were prepared. Cell lysates were subjected to SDS‐PAGE and Western blotting. Specific antibodies against NKX2‐5, TEAD1, TEAD3, phospho‐YAP1, and YAP1 were used. GAPDH served as a housekeeping gene. Densitometry analysis was performed using ImageJ software 31.

**RNA extraction and qPCR.** Total RNA was extracted according to standard protocols using an RNeasy kit (Qiagen) and was then subjected to qPCR analysis using a QuantiFast SYBR Green PCR kit (Qiagen). NKX2‐5 gene expression was normalized against the expression of the β‐actin gene or the TATA box binding protein, as indicated below.

**Immunohistochemistry.** Immunohistochemistry was performed on formalin‐fixed paraffin‐embedded tissue. After antigen retrieval with citric buffer, pH 6.0, sections were immunodecorated with optimally diluted (2 μg/ml in Tris buffered saline [TBS]) antibody NKX2‐5 (catalog no. sc‐14033; Santa Cruz Biotechnology). Biotinylated goat anti‐rabbit secondary antibody (catalog no. BA‐1000; Vector) diluted 2 μg/ml in TBS was used prior to developing with 3,3′‐diaminobenzidine reagent (catalog no. SK‐4100; Vector). Specificity of staining was confirmed using isotype‐matched IgG control antibodies (2 μg/ml).

**Statistical analysis.** Results are reported as the mean ± SEM of data from at least 3 independent experiments. Statistical analyses (Student's unpaired *t*‐test) and graphs were performed using GraphPad Prism 6 software. *P* values less than 0.05 were considered significant.

## Results

**Figure 1:**
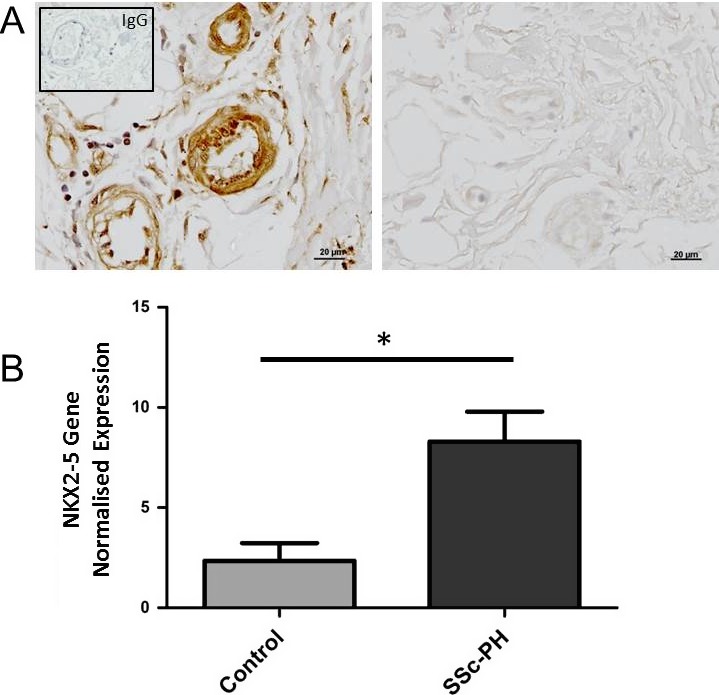
Expression of NKX2‐5 in systemic sclerosis (SSc)–related pulmonary hypertension (PH). **A,** Representative sections of lung tissue from a patient with SSc‐associated PH (of 4 examined) (left) and a healthy control subject (right). Sections were immunodecorated with NKX2‐5 antibody (brown). **Inset,** IgG isotype control. **B,** Normalized expression of the *NKX2-5* gene in pulmonary artery smooth muscle cells isolated from the lung vessels of patients with SSc‐associated PH (n = 5) and healthy control subjects (n = 3). Values are the mean ± SEM. * = *P* < 0.05 by Student's *t*‐test.

**Up-regulation** **of** ***NKX2-5*** **and expression in vascular diseases such as SSc-associated PH.** Initially, *NKX2-5* expression was determined in the pulmonary vasculature of patients with SSc‐associated PH. Immunohistochemical staining revealed that NKX2‐5 was highly expressed in all muscularized arteries in the patients’ lungs, but absent from the vasculature of the healthy subjects’ lungs (Figure 1A). We also used PASMCs isolated from SSc‐associated PH patients and healthy subjects to determine *NKX2-5* messenger RNA (mRNA) expression and found a significant increase (*P* = 0.02) in *NKX2-5* gene expression in the patients (Figure 1B). Our data suggest that *NKX2-5* plays an important role in the vasculature of patients with SSc‐associated PH and that it is regulated at both the gene and protein levels.

Since *NKX2-5* regulation in blood vessels is largely unknown, we investigated the mechanisms at the genetic and transcriptional levels by carrying out a genetic association analysis of the *NKX2-5* locus in SSc patients followed by a functional analysis of the disease‐associated polymorphisms.

**Figure 2:**
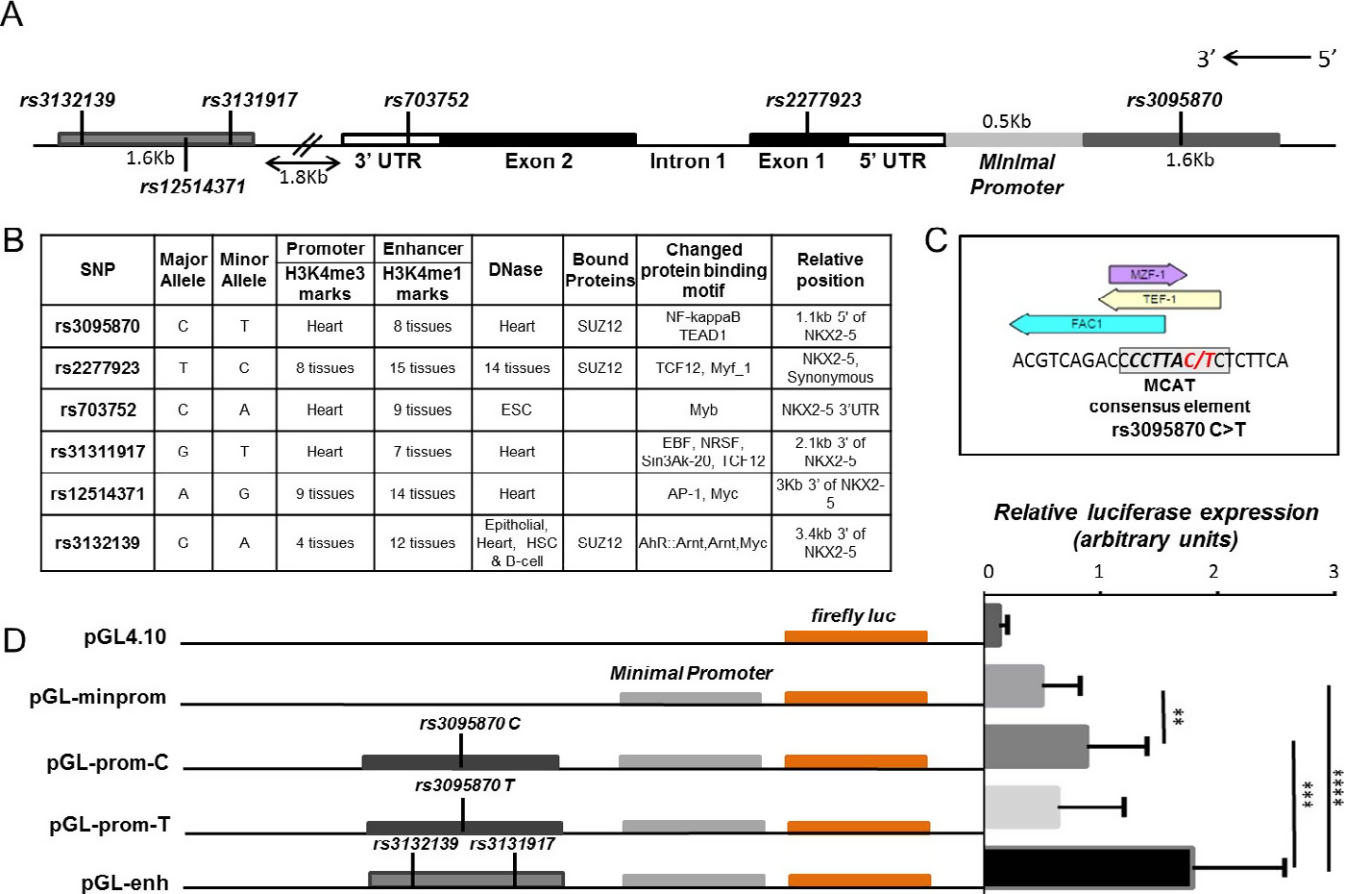
Disease‐associated single‐nucleotide polymorphisms (SNPs) in functional regulatory regions. **A,** Schematic representation of the *NKX2-5* gene with its introns, exons, and untranslated regions (3′‐UTR and 5′‐UTR). The location of the tagging SNPs is shown in the schematic map of the *NKX2-5* genomic locus. **B,** Findings of in silico analysis of the tagging SNPs using HaploReg and Encyclopedia of DNA Elements (ENCODE) data. **C,** Transcription factor binding sites around rs3095870, as determined using the TRANSFAC and JASPAR databases. Boxed area shows the consensus‐binding element for transcription‐enhancer factor domain 1 (TEAD1) on the MCAT site; the alleles of rs3095870 are shown in red. **D,** Transcriptional activity in primary human pulmonary artery smooth muscle cells transfected with reporter gene constructs containing the minimal promoter (pGL‐minprom), upstream promoter with either the rs3095870 C allele (pGL‐prom‐C) or the rs3095870 T allele (pGL‐prom‐T), or the downstream enhancer (pGL‐enh) are shown at the left. The pGL4.10 vector, which contains the firefly luciferase (luc) gene, was used as the cloning vector. The relative luciferase expression for each of the 5 constructs is shown at the right. Values are the mean ± SEM of 3 independent experiments. ** = *P* < 0.01; *** = *P* < 0.001; **** = *P* < 0.0001 by Student's *t*‐test. TCF‐12 = T cell factor 12; ESC = embryonic stem cell; EBF = early B cell factor; NRSF = neuron‐restrictive silencer factor; AP‐1 = activator protein 1; HSC = hematopoietic stem cell; AhR = aryl hydrocarbon receptor; ARNT = aryl hydrocarbon nuclear translocator; MZF‐1 = myeloid zinc finger 1; TEF‐1 = transcriptional enhancer factor 1. Color figure can be viewed in the online issue, which is available at http://onlinelibrary.wiley.com/doi/10.1002/art.40419/abstract.

**Identification of disease-associated SNPs in the** ***NKX2-5*** **locus.** Six tagging SNPs were selected along the *NKX2-5* locus (Figure 2A) and genotyped in 2 independent cohorts of SSc patients (described in detail in Supplementary Tables 1 and 2; available at http://onlinelibrary.wiley.com/doi/10.1002/art.40419/abstract). Meta‐analysis of the 2 cohorts showed association of rs3131917 with SSc (corrected *P* = 0.029, odds ratio [OR] 0.91) (Table 1). Subphenotype association analysis showed that the same SNP was also significantly associated with PH and pulmonary fibrosis in a Spanish cohort (corrected *P* = 0.005 and corrected *P* = 0.04, respectively, OR 0.72 and OR 0.84, respectively) (Table 1), as well as with the anti–RNA polymerase III antibody–positive subgroup in the UK cohort (corrected *P* = 0.023, OR 1.34) (Table 1). Aside from rs3131917, other SNPs within *NKX2-5* were genetically associated with disease subphenotypes. SNP rs3132139 showed a significant association with PH (corrected *P* = 0.002, OR 1.396) and rs3095870 showed a marginal association with pulmonary fibrosis (corrected *P* = 0.04) in the Spanish cohort (Supplementary Table 3, available at http://onlinelibrary.wiley.com/doi/10.1002/art.40419/abstract).

**Table 1:** Genetic association analyses of rs3931917 in a UK and a Spanish cohort of SSc patients via allelic testing^a^

rs3131917	Minor allele	Minor allele frequency		Chi‐square	*P*_corr_ b	OR (95% CI)
		Cases	Controls			
Overall association analysis						
UK, SSc+ vs. SSc–	T	0.48	0.46	1.16	0.28	1.06 (0.94–1.2)
Spanish, SSc+ vs. SSc–	G	0.48	0.51	3.6	0.05	0.9 (0.82–1)
Meta‐analysis					0.029	0.91
Subphenotype analysis						
UK, anti–RNAP III+ vs. control	T	0.54	0.46	6.25	0.023	1.34 (1.06–1.7)
Spanish, PH+ vs. control	G	0.42	0.51	7.8	0.005	0.72 (0.57–0.9)
Spanish, PF+ vs. control	G	0.46	0.51	3.97	0.04	0.84 (0.72–0.99)

a Shown are chi‐square test values, corrected *P* (*P*_corr_) values, and odds ratios (ORs) with 95% confidence intervals (95% CIs) for the genetic association of the rs3131917 single‐nucleotide polymorphism with systemic sclerosis (SSc) in a meta‐analysis of 2 independent cohorts (UK and Spanish), as well as for a subphenotype analysis of the association of rs3131917 with the anti–RNA polymerase III (anti–RNAP III) autoantibody, pulmonary hypertension (PH), and pulmonary fibrosis (PF). The minor allele and its frequencies in cases and controls are also shown. Genetic analyses were performed using Plink.

b *P*_corr_ values for multiple comparisons were determined by permutation analysis.

We further examined the structure of *NKX2-5* using genotype data on the CEU population. The data showed an extended linkage disequilibrium pattern across the region (Supplementary Figure 1A, available at http://onlinelibrary.wiley.com/doi/10.1002/art.40419/abstract), with similar patterns observed in the SSc cohorts (Supplementary Figures 1B and C). The 6 tagging SNPs were merged into 1 haplotype, and an association analysis was performed, which showed a significant association with SSc (*P* ≤ 0.01) (Supplementary Table 4, available at http://onlinelibrary.wiley.com/doi/10.1002/art.40419/abstract). These findings suggest that there is a synergistic, rather than a single‐locus, effect of the SNPs and emphasize the importance of *NKX2-5* in SSc and vascular pathologies as a whole.

**Presence of disease-associated SNPs in potential regulatory regions.** The genetic association study revealed that rs3131917 and rs3132139 were strongly associated with SSc‐associated PH. Since our aim was to determine how *NKX2-5* is activated in diseased vessels, we further explored the potential functional role of the SNPs. Due to the extensive linkage disequilibrium (Supplementary Figure 1), strong associations may mask other functional polymorphisms. Although significantly high *P* values suggest genetic association with disease, they do not necessarily indicate functionality of the associated SNPs. Therefore, using our genetic association data as a guide, we performed in silico studies using HaploReg 26 and ENCODE 23 data to evaluate any potential functional effect of all 6 tagging SNPs (Figure 2B).

SNPs rs3132139, rs3131917, and rs3095870 are located in potential regulatory regions, such as an upstream promoter and a downstream enhancer, based on accumulated evidence from functional annotations, including histone marks specific for promoter and enhancer regions, DNase hypersensitivity sites, and scores for transcription factor binding sites. SNP rs3095870 is located 1.1 kb upstream of the *NKX2-5* transcriptional start site, while rs3131917 and rs3132139 are located 5 kb and 6.4 kb, respectively, downstream of *NKX2-5*, in a region that was previously identified as a putative *NKX2-5* enhancer 32.

First, we looked for transcription factor binding sites that might be affected by the various SNP alleles. Interestingly, we found that rs3095870 is located within a TEAD1 (transcriptional enhancer factor 1 [TEF‐1]) binding site. TEAD1 is a member of the TEA/ATTS domain family transcription regulators with distinct and important roles in VSMC differentiation and in cardiovascular disease 33, 34, 35. All 4 members of the family recognize and bind specifically at the MCAT consensus element: 5′‐TCATTCCT‐3′ (Figure 2C). The minor T allele of rs3095870 disrupts the consensus sequence and could obliterate the TEAD1 binding site (Figure 2C). This suggests that TEAD1 might regulate *NKX2-5* expression, depending on the presence of the rs3095870 C allele. The rs3095870 C allele showed a marginal association with pulmonary fibrosis (corrected *P* = 0.04) in the Spanish cohort (Supplementary Table 3).

We investigated the effect of the C/T alleles of rs3095870 and the other associated SNPs on *NKX2-5* transcriptional activity using luciferase reporter assays. A 1.6‐kb genomic region containing the rs3095870 C allele was cloned into a pGL4.10 reporter vector (pGL‐prom‐C) driven by the *NKX2-5* minimal promoter (pGL‐minprom). The alternative T allele was introduced by site‐directed mutagenesis (pGL‐prom‐T), and the constructs were transfected into primary human PASMCs. Addition of the 1.6‐kb upstream region increased the relative expression of luciferase as compared to the minimal promoter, but only in the presence of the rs3095870 C allele (*P* < 0.01) (Figure 2D). These data suggest that rs3095870 is a functional SNP that requires further investigation.

When a 1.6‐kb genomic region downstream of *NKX2-5*‐containing SNPs rs3132139 and rs3131917 was cloned next to the minimal promoter (pGL‐enh), luciferase expression was increased significantly compared to both pGL‐prom‐C and pGL‐prom‐T (*P* < 0.0001) (Figure 2D). The data demonstrate that the 3 associated SNPs are indeed present within functional regulatory elements that directly influence *NKX2-5* transcriptional activity, and they confirm that the region containing rs3132139 and rs3131917 is an active enhancer.

**Figure 3:**
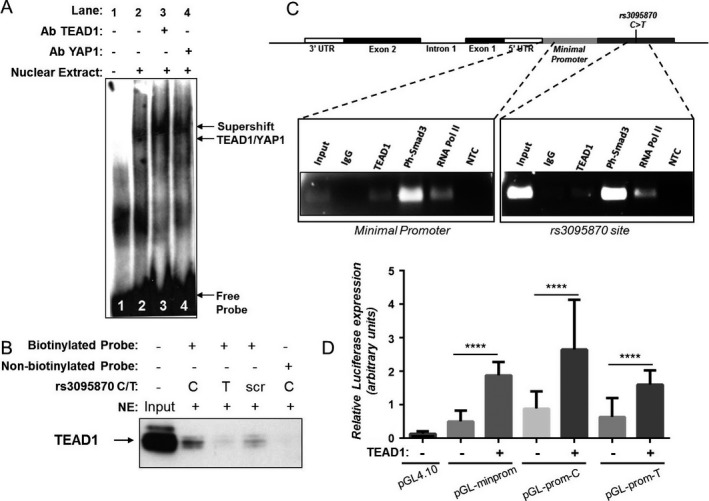
Binding of transcription‐enhancer factor domain 1 (TEAD1) at the rs3095870 site and increased *NKX2-5* transcriptional activity. Binding assays were performed using nuclear extracts (NE) from transforming growth factor β–treated immortalized human pulmonary artery smooth muscle cells (PASMCs) and biotinylated DNA probes containing the rs3095870 C/T alleles (**A–C**). **A,** Electrophoretic mobility shift assay with rs3095870 C allele biotinylated probe (lane 2), and supershifts with TEAD1 and Yes‐associated protein 1 (YAP1) antibodies (Ab) (lanes 3 and 4), as well as without nuclear extract (lane 1). **B,** Pull‐down assay using streptavidin beads and biotinylated DNA probes specific to rs3095870 C/T alleles. Complexes were analyzed by sodium dodecyl sulfate–polyacrylamide gel electrophoresis and Western blotting and immunodecorated with TEAD1 antibody. Nonbiotinylated and scrambled (scr) biotinylated probes were used as controls. Input was 5% of total nuclear extract. **C,** Chromatin immunoprecipitation assays using antibodies specific for TEAD1, phosphorylated Smad3 (Ph‐Smad3), and RNA polymerase II (RNA Pol II). Specific polymerase chain reaction primers were designed and used to detect enrichment. Immunoprecipitation with IgG was used as a control. No template control (NTC) was also used. Input was 10% of the initial chromatin used per immunoprecipitation assay. A schematic representation of the *NKX2-5* gene, showing intron 1, exons 1 and 2, and untranslated regions (3′‐UTR and 5′‐UTR), as well as the locations of the minimal promoter and rs3095870 C>T site, is shown at the top. **D,** Luciferase reporter gene assays of primary human PASMCs. TEAD1 expression vector was cotransfected with DNA constructs containing either the minimal promoter (pGL‐minprom) or the upstream promoter with the rs3095870‐C/T alleles (pGL‐prom‐C/T). The pGL4.10 vector, which contains the firefly luciferase gene, was used as the control. Values are the mean ± SEM of 3 independent experiments. **** = *P* ≤ 0.0001 by Student's *t*‐test.

**TEAD1 regulation of** ***NKX2-5*** **transcription through binding on rs3095870.** To corroborate the reporter gene data and the effect of TEAD1 binding on *NKX2-5* gene expression, we conducted DNA–protein binding and ChIP assays. TEAD1 specifically binds DNA on MCAT elements, but it also requires cofactors to exert its activity. YAP1 has been identified as the best candidate for TEAD‐dependent transcription 36, 37. Using EMSA, we found that different bands were formed for each allele, suggesting that nuclear protein binding was different for the C and T alleles of rs3095870 (Supplementary Figure 2, available at http://onlinelibrary.wiley.com/doi/10.1002/art.40419/abstract). Supershift assays confirmed that TEAD1 and YAP1 are both components of the rs3095870 C–binding complex (Figure 3A).

To provide further evidence, we performed protein pull‐down assays using biotinylated DNA probes containing either the C or the T allele of rs3095870. The assays demonstrated that TEAD1 specifically binds the C allele of the SNP (Figure 3B). In addition, ChIP assays confirmed TEAD1 binding in the region upstream of the *NKX2-5* transcriptional start site in transforming growth factor β (TGFβ)–treated immortalized human PASMCs (Figure 3C). Interestingly, ChIP assay analysis revealed strong enrichment for phosphorylated Smad3 protein on the *NKX2-5* upstream region (Figure 3C), verifying the involvement of TGFβ in *NKX2-5* regulation, which has previously been reported 12. Although enriched binding for both TEAD1 and phosphorylated Smad3 antibodies was observed on the minimal *NKX2-5* promoter, the upstream rs3095870 region was also immunoprecipitated despite the strong input signal. The finding that RNA polymerase II also precipitated the same region (Figure 3C) suggests that TEAD1 and phosphorylated Smad3 are engaged in the transcriptional machinery bound to the minimal promoter.

We investigated a potential mechanism of *NKX2-5* transcriptional activation by TEAD1. As expected, when TEAD1 expression vector was cotransfected into human PASMCs together with the pGL‐prom‐C/T constructs, the transcriptional activity was strongly increased (*P* < 0.0001) (Figure 3D). Taken together, these data indicate a mechanism of transcriptional regulation through the specific binding of TEAD1 on the C allele of rs3095870 in the upstream promoter region of *NKX2-5*.

**Figure 4:**
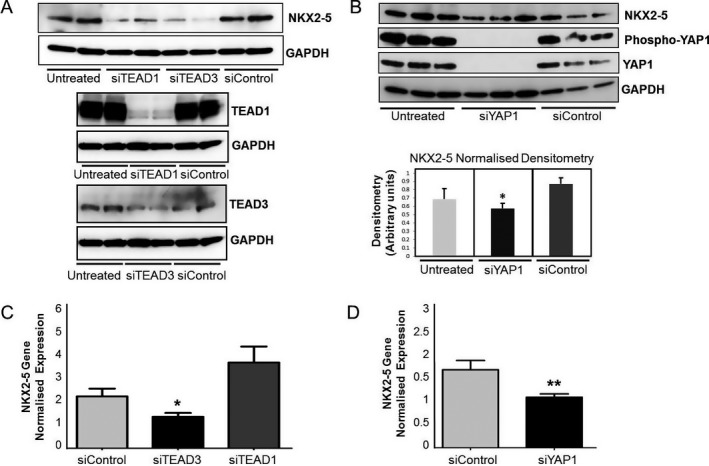
NKX2‐5 transcriptional regulation through transcription‐enhancer factor domain 1 (TEAD1)/Yes‐associated protein 1 (YAP1) complexes. **A,** NKX2‐5 expression in immortalized human pulmonary artery smooth muscle cells (PASMCs), as determined by sodium dodecyl sulfate–polyacrylamide gel electrophoresis (SDS‐PAGE) and Western blotting after TEAD1 and TEAD3 protein knockdown using small interfering RNA (siRNA)–specific oligonucleotides (siTEAD1 and siTEAD3). To evaluate the efficiency of silencing, TEAD1 and TEAD3 protein levels were also determined by SDS‐PAGE and Western blotting. Scrambled nontargeting siRNA molecules were used as a control (siControl), and GAPDH was used as a housekeeping gene. **B,** Protein levels of NKX2‐5, phosphorylated YAP1, and YAP1 in immortalized human PASMCs, as determined by SDS‐PAGE and Western blotting after YAP1 knockdown using specific siRNA (siYAP1) (top). Protein levels of NKX2‐5 were then analyzed by densitometry, and the normalized levels are shown at the bottom. Values are the mean ± SEM of 3 independent experiments. **C** and **D,** *NKX2-5* gene expression in total RNA isolated from immortalized human PASMCs after treatment with siTEAD1 or siTEAD3 (**C**) or with siYAP1 (**D**), as determined by quantitative polymerase chain reaction analysis. Scrambled siControl was used for comparison, and *NKX2-5* gene expression was normalized against levels of TATA‐binding protein (housekeeping gene). Values are the mean ± SEM of 3 independent experiments. * = *P* ≤ 0.05; ** = *P* ≤ 0.01 by Student's *t*‐test.

We next investigated whether knockdown of TEAD1 using siRNA affects NKX2‐5 expression in immortalized human PASMCs. Indeed, we found that NKX2‐5 protein levels were decreased when TEAD1 was knocked down (Figure 4A), but there was no statistically significant change in the mRNA levels (Figure 4C). Since all the TEAD family members recognize and bind the same DNA consensus element, we wanted to determine whether another member of the family may also be implicated in the regulatory mechanism. A recent study showed that TEAD3 is expressed in human aortic SMCs and is required for the TGFβ signaling cascade 38, and we therefore investigated whether TEAD3 can also regulate *NKX2-5*. When TEAD3 was knocked down by siRNA, the gene and protein levels of NKX2‐5 were significantly decreased (Figures 4A and C). However, when immortalized human PASMCs were cotransfected with TEAD3 expression vector together with the pGL‐prom‐C/T constructs, the luciferase activity was not altered (Supplementary Figure 3, available at http://onlinelibrary.wiley.com/doi/10.1002/art.40419/abstract). We next verified that YAP1 is a cofactor for TEAD1/3 proteins by showing that NKX2‐5 gene and protein levels were significantly decreased when YAP1 was knocked down using siRNA (Figures 4B and D).

Taken together, these data suggest a complex regulatory role of TEAD1 and TEAD3. The data are consistent with a transcriptional mechanism whereby TEAD3 is responsible for basal levels of expression and TEAD1 is required for higher activation. At the same time, it is possible that they both control factors that regulate NKX2‐5 at the posttranscriptional and posttranslational levels. Regardless, the data confirm that the TEAD site at rs3095870 is functional and that binding of TEAD/YAP1 regulates NKX2‐5 expression in human PASMCs.

**Transcriptional regulation of** ***NKX2-5*** **through the downstream enhancer.** The rest of the disease‐associated SNPs (rs3132139, rs3131917) are located downstream of *NKX2-5*. This region was previously described as a candidate cardiac enhancer sequence due to its proximity to the *NKX2-5* gene, although it has not yet been verified experimentally 32. In this study, we used luciferase reporter gene assays to confirm that the region is indeed a *NKX2-5* enhancer in human PASMCs (Figure 2D).

To obtain more information on the transcription factors that can potentially interact with this enhancer and the 2 SNPs in particular, we conducted another in silico analysis. It revealed several binding sites for a number of DNA binding proteins known to regulate transcription through enhancer regions during heart development and SMC differentiation, such as GATA‐6, myocyte‐specific enhancer factor 2C (MEF‐2C), and c‐Jun. Since enhancer effects are usually cell type–specific, we performed ChIP assays in immortalized human PASMCs.

**Figure 5:**
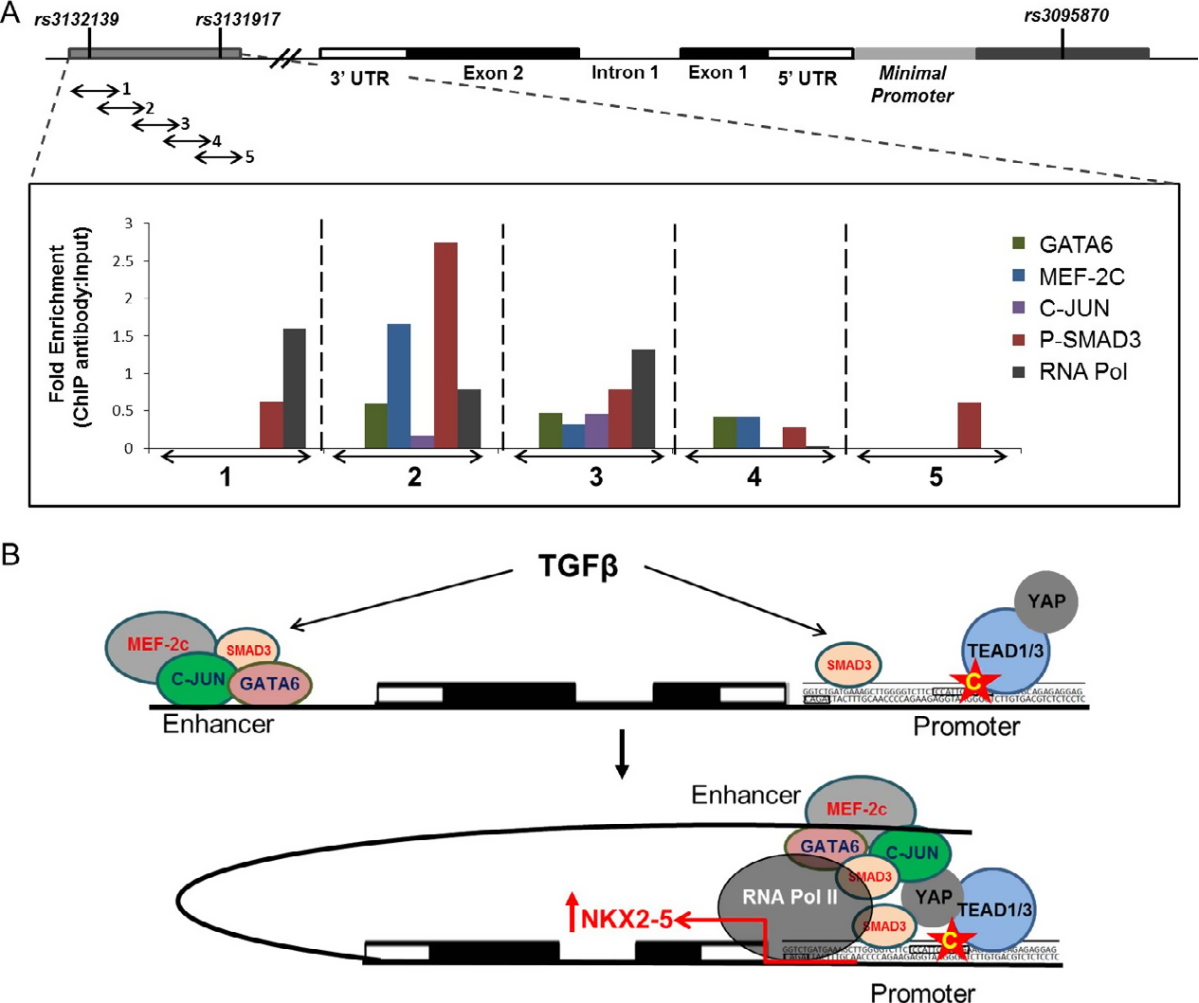
An upstream promoter and a downstream enhancer involved in NKX2‐5 transcription. **A,** Analysis of the enrichment of GATA‐6, myocyte‐specific enhancer factor 2C (MEF‐2C), c‐Jun, phosphorylated Smad3, and RNA polymerase II (RNA Pol) as determined by chromatin immunoprecipitation (ChIP) assays of transforming growth factor β (TGFβ)–treated immortalized human pulmonary artery smooth muscle cells. Chromatin was immunoprecipitated using specific antibodies. Five primer pairs 1, 2, 3, 4, 5 were designed to cover the 1.6‐kb genomic region of the downstream enhancer. Results are shown as the fold enrichment of each protein in every genomic segment. The data were normalized against the intensity of the input (10% of initial chromatin used per immunoprecipitation assay). A schematic representation of the *NKX2-5* gene, showing intron 1, exons 1 and 2, and untranslated regions (3′‐UTR and 5′‐UTR), as well as the locations of rs3132139, rs3131917, rs3095870, and the minimal promoter sites, is shown at the top. **B,** Proposed model of the transcriptional regulation of *NKX2-5*. Functional studies revealed an upstream promoter region and a novel downstream functional enhancer that are engaged in the transcriptional initiation machinery of *NKX2-5* through the binding of TEAD1/TEAD3/YAP1 complex and other transcription activators, such as MEF‐2C, c‐Jun, phosphorylated Smad3, and GATA‐6. **Red arrow** to the right of *NKX2-5* is the transcription start site. Star with the letter C indicates the C allele of rs3095870. Color figure can be viewed in the online issue, which is available at http://onlinelibrary.wiley.com/doi/10.1002/art.40419/abstract.

Surprisingly, we found significant enrichment for MEF‐2C, GATA‐6, and c‐Jun at the 5′ end of the enhancer region (Figure 5A). Phosphorylated Smad3 protein showed significant binding across the entire region, suggesting that TGFβ could also activate the enhancer to positively regulate *NKX2-5* expression (Figure 5A). In addition, an interesting finding was the RNA polymerase II enrichment toward the 5′ end of the region, suggesting that the enhancer is engaged with the transcriptional machinery through an enhancer–promoter interaction (Figure 5A). These data confirm that the downstream genomic locus, which contains the disease‐associated SNPs, is a functional enhancer that activates *NKX2-5* transcription in human PASMCs through the binding of GATA‐6, MEF‐2C, and c‐Jun.

## Discussion

In this study, we demonstrated the molecular mechanisms that underlie the overexpression of NKX2‐5 in SSc. We also identified potential genetic associations with functional polymorphisms that may contribute to SSc susceptibility and specifically to the important vascular complication of SSc‐associated PH.

Recent published work proposes that modulated NKX2‐5 expression is implicated in vascular homeostasis and vascular disease–associated phenotypes 39, 40, 41. In our previous studies, we demonstrated a critical role of NKX2‐5 in vascular remodeling and atherosclerosis, with expression being detected in atherosclerotic lesions, in the fibrous cap, and in the cells within the media 5, 6. However, the mechanisms that lead to up‐regulation of NKX2‐5 expression in vessels remain unknown. In the present study, we performed candidate gene association analysis to explore our evidence‐based hypothesis that *NKX2-5* is genetically associated with vascular disease characterized by vascular remodeling as well as to investigate the functional effect of any associated loci. Despite the numerous reports of genomic data either published or stored in repositories, limited information is available on *NKX2-5*, presumably due to the perception that the role of the gene is important exclusively in embryonic development 1, 2, 3, 4.

Autoimmunity and dysregulation of immune responses are well‐established components of SSc, and the major histocompatibility complex has been predominantly associated with SSc by the majority of genetic studies and genome‐wide association studies 42, 43. However, outside autoimmunity, only a few genes have been associated with SSc, with *CTGF* (*CCN2*) being the most studied 44, 45. We performed a genetic association study in 2 independent cohorts of SSc patients as a model of vascular and pulmonary pathology. Interestingly, in the meta‐analysis, we found rs3131917 to be associated with SSc (*P* = 0.029). This is a de novo finding of an association between SSc and a gene unrelated to immunity in a meta‐analysis across 2 independent cohorts of similar origin.

The groups of SSc patients with pulmonary complications such as PH and pulmonary fibrosis have the highest mortality rates 46. We found that rs3132139 and rs3131917 are genetically associated with PH (Table 1 and Supplementary Table 3). These data support our hypothesis that the *NKX2-5* genomic locus is important in pulmonary/vascular pathology. In fact, rs3132139 showed a significant association (*P* = 0.006) in a meta‐analysis of 46 genome‐wide association studies in patients with coronary artery disease (CAD) 47. CAD is an arterial disease characterized by plaque formation and constrictive vascular remodeling in the carotid artery, as well as endothelial dysfunction and inflammation 48. This finding further supports our hypothesis for the functional involvement of NKX2‐5 in vascular remodeling.

Surprisingly, both rs3131917 and rs3132139 reside in a region that was identified as a putative enhancer (chromosome 5: 173,228,601–173,230,244) for *NKX2-5* in heart tissue by ChIP‐Seq analysis 32. This enhancer should be activated when NKX2‐5 exerts its unique and nonredundant role during heart development. We hypothesized that the same mechanism that activates NKX2‐5 in embryogenesis could also activate the gene in diseased vessels in adulthood. In this study, we validated the region as a functional downstream *NKX2-5* enhancer. In reporter gene assays, luciferase activity was significantly increased in the presence of this enhancer compared to the proximal minimal promoter. We have shown that there is enriched binding of the transcription factors MEF‐2C, GATA‐6, and c‐Jun on this enhancer. This result supports early studies showing that MEF‐2C/NKX2‐5/GATA form a positive regulatory network 12, 49, 50. Further studies are being carried out on this enhancer in order to characterize its precise function.

We also identified TEAD1 as a transcription regulator for *NKX2-5* activation in human PASMCs. TEAD1 can only bind the *NKX2-5* upstream promoter at –1.1 kb in the presence of the rs3095870 C allele. TEADs are expressed in VSMCs, where they are known to control phenotype modulation from the contractile to the synthetic state, as well as cell proliferation and the cell cycle 33, 34. We demonstrated that TEAD1 bound strongly on the upstream promoter at the rs3095870 C allele; however, it did not significantly affect *NKX2-5* mRNA in siRNA knockdown experiments. On the contrary, specific siRNA for TEAD3 significantly decreased both the protein and RNA levels of NKX2‐5. TEAD3 cotransfection in human PASMCs, however, did not increase luciferase expression.

These data indicate a complex transcriptional mechanism that involves both TEAD1 and TEAD3. One explanation could be that TEAD3 is directly required for basal levels of *NKX2-5* transcription through binding on another MCAT element located outside the 1.6‐kb upstream region containing rs3095870 (chromosome 5: 173,235,792–173,237,444). In the presence of TEAD1, however, the transcription is significantly enhanced (Figure 3D), as TEAD1 is required for the overexpression of *NKX2-5* by binding to the promoter containing rs3095870 in response to injury or disease‐associated stimuli.

A recent study showed that TEAD3 is required for TGFβ signaling through association with Smad3 in human aortic SMCs in a risk locus for atherosclerosis 38. Our data add to the accumulating evidence of the emerging roles of TEADs and TGFβ signaling in VSMCs in cardiovascular disease. The four members of the TEAD family recognize the same MCAT binding element, and they require cofactors in order to regulate the expression of downstream targets. YAP1 is the most common associating partner of TEADs, especially in VSMCs 36, 37. Indeed, we were able to confirm that YAP1 is important for NKX2‐5 activity, corroborating the role of TEAD/YAP1 in *NKX2-5* transcriptional regulation.

We propose a new mechanism for the regulation of the *NKX2-5* gene in human PASMCs, which is described in detail in Figure 5B. The mechanism involves an upstream promoter activated through the binding of the TEAD/YAP1 complex. This promoter is engaged with the RNA polymerase II complex, and a downstream enhancer that binds GATA‐6, MEF‐2C, and c‐Jun. Enrichment of phosphorylated Smad3 binding confirmed that TGFβ, which is known to activate NKX2‐5 expression, exerts its effect on NKX2‐5 through Smad3 binding at multiple CAGA sites on both the upstream promoter and the downstream enhancer. Evidence from the ChIP assays clearly shows that both regions interact with the RNA polymerase II complex and the transcriptional machinery. Detailed studies will be necessary to further investigate a potential combined synergistic effect of the upstream promoter and the downstream enhancer on the transcriptional activity of *NKX2-5* in human PASMCs. Transcriptional activation, and therefore NKX2‐5 expression, in VSMCs results in the up‐regulation of NKX2‐5 downstream targets associated with inflammation, proliferation, migration, extracellular matrix production, and deposition within the vascular wall, all of which are key features of vascular remodeling and ultimately lead to disease‐associated pathogenic phenotypes 5, 6.

In summary, we have shown that *NKX2-5* is genetically associated with SSc and PH, and we propose for the first time a regulatory mechanism for the transcriptional activation of the human *NKX2-5* gene in diseased vessels. The mechanism involves an upstream promoter that binds the TEAD/YAP1 complex and a novel enhancer activated through the binding of GATA‐6, MEF‐2C, and c‐Jun. We believe that this is a key mechanism that is common in conditions characterized by different types of vascular remodeling, including but not limited to PAH, CAD, peripheral artery disease, and stroke.

## Author contributions

All authors were involved in drafting the article or revising it critically for important intellectual content, and all authors approved the final version to be published. Dr. Ponticos had full access to all of the data in the study and takes responsibility for the integrity of the data and the accuracy of the data analysis.

### Study conception and design

Dritsoula, Martin, Herrick, Abraham, Denton, Ponticos.

### Acquisition of data

Dritsoula, Ponticos.

### Analysis and interpretation of data

Dritsoula, Papaioannou, Guerra, Fonseca, Ponticos.
