# Supplementary material for: Molecular Basis for Dysregulated Activation of NKX2‐5 in the Vascular Remodeling of Systemic Sclerosis
Source: Arthritis Rheumatol. 2018 Apr 24;70(6):920–31. doi: 10.1002/art.40419 (PMC6001790; doi:10.1002/art.40419)
Supplement: Supplementary file 1 [file ART-70-920-s001.docx]

**SUPPLEMENTARY DATA**

**Methods and materials**

Data on coronary artery disease/myocardial infarction have been contributed by CARDIoGRAMplusC4D investigators and have been downloaded from [www.CARDIOGRAMPLUSC4D.org](http://www.CARDIOGRAMPLUSC4D.org).

**Immortalised HPASMC**

The cell line (Cat. no: T0558, ABM Good) was immortalised via a lentivirus containing SV40 and underwent QC control for transgene expression. The lentiviral vector map and the construct sequence can be found here <https://www.abmgood.com/pLenti%20SV40-Vector-Properties.html>. The morphology, growth rate, response to cytokines/growth factors and expression profile of the immortalised cells were compared and found similar to that of primary cells prior to use in experimental techniques.

**Immunohistochemistry.** Endogenous peroxidase was blocked using 3% hydrogen peroxide. NKX2-5 staining was visualized by DAB and sections were counterstained by hematoxylin. Sections were examined with an Axioskop Z bright field microscope (Carl Zeiss, Germany) using Axiovision software.

**Taqman Genotyping.** 20ng of DNA in a 5µl total reaction was used for PCR reaction on an ABI GeneAmp PCR System 9700 according to the manufacturer’s instructions. For the post-read and the allelic discrimination protocols an ABI Prism 7900HT Real-Time Thermocycler was used. High Resolution Melting (HRM) analysis. To amplify a 85bp region surrounding rs3095870, 10-20ng of DNA was used in a 10µl total reaction with 0.7µM of each of the following primers: Forward: 5’- gactcctgaattgtaagcaa-3’, Reverse: 5’- gggaggtctgatgaaagc-3’. The DNA amplification was done in a Corbett Rotor Gene 6000 cycler.

**Cloning and Luciferase Assays.** A 578bp fragment (chr5: 173,235,215-173,235,791) upstream of the NKX2-5 transcription start site encompassing the minimal promoter of the gene was amplified from a DNA sample of a healthy donor and cloned into the pGL4.10 reporter vector (Promega) prior to the Firefly luciferase gene (pGL-minprom). A longer fragment of 1.6Kb (chr5:173,235,792-173,237,444) upstream of NKX2-5 minimal promoter including the minor T allele of SNP rs3095870 was also amplified and cloned into the pGL4.10 vector upstream of the minimal promoter (pGL-prom-T). Site directed mutagenesis (Quick Change II, Agilent) was used to introduce the major C allele of the SNP (pGL-prom-C). For the analysis of the putative enhancer, a genomic locus of 1.6Kb (Chr5: 173,228,601-173,230,244) downstream of NKX2-5 that contained the two associated polymorphisms (rs3132139, rs3131917) was amplified in a similar way and cloned into the pGL4.10 reporter vector near the minimal promoter (pGL-enh). The integrity of the cloned sequences and the backbone of the vectors were verified by sequencing. For the luciferase assays, primary HPASMC were seeded in 24-well plates at 70.000-80.000 cells/well and transfected with constructs as indicated to perform luciferase assays. Transfection mixtures were prepared with Fugene HD transfection reagent in Optimem (Gibco) at a ratio of 3:1 (transfection reagent to DNA). A maximum of 825ng DNA was added per well. Cells were cultured for 48 hours before they were lysed and subjected to analysis for luciferase expression. Luciferase activity was measured using the Dual-Glo Luciferase Assay System (Promega) in a Mithras LB 940 Plate Reader. The results represent the ratios of Firefly/Renilla luciferase expression and are the mean of at least 3 independent experiments.

**RNA Silencing.** ImHPASMC were plated in 12-well plates at 60% confluence and serum-starved overnight. Next morning, the cells were transfected with 130nM of siRNA for YAP1 and 100nM of siRNA for TEAD1 and TEAD3 complexed with Oligofectamine (Invitrogen) in serum free media. Four hours after transfection serum-supplemented media was added to the cells to a final concentration of 10%. 48 hours after transfection of YAP1 and 72hours after transfection of TEAD1 and TEAD3 the cells were collected for RNA and protein extraction. RNA and protein levels of TEAD1, TEAD3, YAP1 and NKX2-5 were assessed by qPCR and western blotting.

# Binding Assays

For the binding assays two 54bp-biotinylated double-stranded DNA probes were designed spanning the rs3095870 site. Each probe was specific to either the C or the T allele of the SNP. For the pull down assays two negative controls were used: a 54bp-biotinylated probe with scrambled DNA sequence and a non-biotinylated probe specific to the C allele of rs3095870.

**Electromobility Shift Assays (EMSA).** Immortalised HPASMC were used to prepare nuclear extracts (NE-PER extraction kit, Thermo Scientific). 4µg of nuclear extract and 20fmol of each biotinylated DNA probe were added in a total volume of 20μl binding reaction (10mM Tris, 150mM KCl, 1mM DTT, 10% Glycerol, 15mM MgCl_2_, 200ng dI-dC, 0.05% NP-40). For the super-shifts, 1-2μg of antibodies were used per reaction (TEAD1, ab133533, Abcam; YAP1,). The reactions were incubated for 20 minutes at room temperature and then loaded into a 6% native polyacrylamide gel. The gel ran in 0.5x TBE at 120V for 3 hours at 4^o^C. The DNA: protein complexes were transferred to a positive nylon membrane for 30 minutes at 15V using a Trans-blot semi-dry apparatus (Biorad). The membrane was UV-light crosslinked for 1 minute (UV Stratalinker 2400, Stratagene). Then, the membrane was probed with streptavidin-HRP conjugate, incubated with the substrate and developed following a standard protocol (LightShift™ Chemiluminescent EMSA Kit, ThermoFisher).

**Pull Down assays.** Immortalised HPASMC were serum-starved in 0.1%FBS in DMEM for six hours before treated with TGF-β for 16 hours. The cells were washed twice in ice-cold PBS and scraped with cell scrapers in ice-cold Hank’s balanced salt solution (HBSS, Gibco). The cell suspension was spun down at 4^o^C and the cell pellet was used to prepare nuclear extracts (NE-PER extraction kit, Thermo Scientific). 0.5nmol of each DNA probe was added in 1ml of binding buffer (10mM HEPES, 0.5mM EDTA, 0.5mM DTT, 10% Glycerol, 200ug Salmon sperm DNA) together with 100µl streptavidin-coated paramagnetic beads (Promega™ Streptavidin MagneSphere™ Paramagnetic Particles). The beads had previously been washed three times in PBS and blocked in 200μg BSA in PBS for one hour. The DNA and the beads were rotated at 4^o^C for one hour. Then ~60-70μg of nuclear extract supplemented with protease and phosphatase inhibitors were added in each binding reaction and rotated at 4^o^C for one hour. The protein:DNA:beads complexes were washed five times for five minutes each wash in 150mM NaCl, 10mM HEPES, 0.1% NP-40. The proteins were eluted from the complexes in LDS sample buffer and reducing agent (Thermo Fisher) and were loaded in precast polyacrylamide gels (Thermo Fisher) for western blotting.

**ChIP assays.** Immortalised HPASMC were serum-starved in 0.1%FBS in DMEM for six hours before treated with TGF-β for 16 hours. Then, the cells were cross-linked for 10 minutes with1% formaldehyde at 37^o^C. The cells were washed twice with ice-cold PBS supplemented with protease inhibitors and then lysed directly into SDS lysis buffer (1% SDS, 10mM EDTA, 50mM Tris pH8.1, protease inhibitors) using cell scrapers. The cell suspension was transferred into 2ml-tubes and kept on ice for 15 minutes. The chromatin was sonicated to a mean size of 300–500bp. Lysed cells were cleared by centrifugation at maximum speed for 10 minutes at 4^o^C and then immunoprecipitation assays were followed based on a standard protocol (Upstate, Millipore). 2–4μg of antibodies (Rabbit IgG, sc-2027, Santa Cruz; TEAD1, ab133533, Abcam; RNA polymerase II, sc-899, Santa Cruz; phospho-Smad3, 9520, Cell Signalling, GATA6, sc-9055, Santa Cruz; C-Jun, sc-1694, Santa Cruz; Mef-2c, sc-13268, Santa Cruz) were added in each reaction and incubated overnight rotating at 4°C. To reverse cross-linking the eluates were incubated overnight at 65°C after the addition of 80mM NaCl. The next day, 4mM EDTA, 16mM Tris-HCl and proteinase K were added to the eluates and incubated at 45^o^C for one hour. The DNA was phenol:chloroform extracted and ethanol precipitated in the presence of glycogen. Purified DNA was further subjected to standard PCR (primers available in the supplementary data).

**Monoclonal antibodies.** TEAD1 (ab133533, Abcam), TEAD3 (13224, Cell Signalling), phospho-YAP1 (4911, Cell Signalling), YAP1 (4912, Cell Signalling), NKX2-5 (ab54567, Abcam), GAPDH (ab8245, Abcam).

**Human qPCR primers.** NKX2-5: forward 5’-GAGCCGAAAAGAAAGAGCTGTG-3’ and reverse 5’-GGAACCAGATCTTGACCTGCGT-3’. TATA-binding protein (TBP): forward 5’- AGTGACCCAGCATCACTGTTT-3’ and reverse 5’- GGCAAACCAGAAACCCTTGC-3’).

**Supplementary Table 1.**

| **Sub-phenotypes** | **UK Cohort** | **Spanish Cohort** |
| --- | --- | --- |
| **Gender** |  |  |
| **Female** | 1124 (84.25%) | 1504 (86.6%) |
| **Male** | 210 (15.74%) | 188 (10.8%) |
| **NA** |  | 44 |
| **Disease Subtype** |  |  |
| **LcSSc** | 945 (70.9%) | 1034 (64.5%) |
| **DcSSc** | 387 (29%) | 428 (26.7%) |
| **Overlap** | 2 | 23 (1.43%) |
| **Auto-Antibodies** |  |  |
| **ACA** | 472 (35.38%) | 735 (42.33%) |
| **ATA** | 253 (18.9%) | 351 (20.21%) |
| **ARA** | 172 (12%) | NA |
| **Organ Involvement** |  |  |
| **PF** | 467 (35%) | 388 (22.3%) |
| **PH** | 195 (12.36%) | 170 (9.8%) |
| **RC** | 78 (5.8%) | NA |
| **Total Cases** | **1334** | **1736** |
| **Total Controls** | **901** | **1753** |

**Supplementary Table 1.** **Description of Cohorts.** The patients were categorised in groups based on the presence of auto-antibodies, organ involvement and the SSc subsets. Auto-antibody groups: ATA: anti-topoisomerase; ACA: anti-centromere; ARA: anti-RNA polymerase. Organ involvement: PF: Pulmonary fibrosis; PAH: Pulmonary arterial hypertension; PH: Pulmonary Hypertension; RC: Renal crisis. SSc subsets: lcSSc: limited SSc, dcSSc: diffuse SSc.

**Supplementary Table 2. Hardy-Weinberg equilibrium in the two SSc cohorts**

| **SNP** | **Minor** | **Major** | **Cohort** | **Genotype Counts** | **Observed Heterozygosity** | **Expected Heterozygosity** | **Hardy-Weinberg p** |
| --- | --- | --- | --- | --- | --- | --- | --- |
| rs3132139 | A | G | UK | 95/417/364 | 0.476 | 0.4529 | 0.1364 |
|  |  |  | Spain | 201/816/702 | 0.4747 | 0.4575 | 0.1265 |
| rs12514371 | C | T | UK | 23/255/598 | 0.2911 | 0.2846 | 0.554 |
|  |  |  | Spain | 46/469/1204 | 0.2728 | 0.2731 | 0.9298 |
| rs3131917 | G | T | UK | 190/448/248 | 0.5056 | 0.4979 | 0.6856 |
|  |  |  | Spain | 392/847/358 | 0.5304 | 0.4998 | 0.01622 |
| rs703752 | A | C | UK | 102/416/346 | 0.4815 | 0.4601 | 0.1838 |
|  |  |  | Spain | 154/819/761 | 0.4723 | 0.4387 | 0.00149 |
| rs2277923 | G | A | UK | 60/388/428 | 0.4429 | 0.4118 | 0.0268 |
|  |  |  | Spain | 157/736/799 | 0.435 | 0.428 | 0.5322 |
| rs3095870 | T | C | UK | 102/427/338 | 0.4925 | 0.463 | 0.06671 |
|  |  |  | Spain | 142/732/687 | 0.4689 | 0.4391 | 0.00796 |

**Supplementray Table 3**

| **Sub-phenotype analysis** |  | **Test** | **Minor Allele** | **MAF in Cases** | **MAF in Controls** | **x^2^** | ***p**(corrected)** | **OR (95% CI)** |
| --- | --- | --- | --- | --- | --- | --- | --- | --- |
| rs3132139 | PH^+^ Vs Control | Allelic | A | 0.43 | 0.35 | 8.28 | 0.002 | 1.396 (1.11-1.75) |
| rs3095870 | PF^+^ Vs Control | Dominant TT + TC Vs CC | T | 0.29 | 0.32 | 5.25 | 0.04 |  |

**Supplementary Table 3. Sub-phenotype analysis in the Spanish cohort.** SNPs in the NKX2-5 genomic locus are genetically associated with pulmonary hypertension (PH) and pulmonary fibrosis (PF) in the Spanish SSc cohort. The chi-squared test values (x2), the ps (p*), the odds ratios (OR) and the 95% confidence interval (95% CI) for the allelic test of rs3132139 and the dominant test of rs3095870 are shown. The given p-values (p*) are corrected for multiple comparisons using permutation analysis. The genetic analysis was performed in Plink.

**Supplementary Table 4.**

| **SNPs in the cohort** | **Cohort** | **Haplotype** | **Frequency of haplotype (Cases/Controls)** | **x^2^** | **p** |
| --- | --- | --- | --- | --- | --- |
| rs3132139\| rs12514371\| rs3131917\| rs703752\| rs2277923\| rs3095870 | UK | GTTAAA | 0.015/ 0.004 | 13.6 | <0.01 |
|  | Spanish | GTTCAG | 0.013/ 0.008 | 5.63 | 0.01 |

**Supplementary Table 4**. **Haplotype association analysis in the two independent cohorts**. The six tagging SNPs along the *NKX2-5* genomic locus were pooled together to form a haplotype, and a haplotype association test was performed in Haploview. The haplotypes formed were similar in both populations and also showed evidence of association (p≤0.01). However, these haplotypes do not physically occur in the population.

**Supplementary Figure 1.**


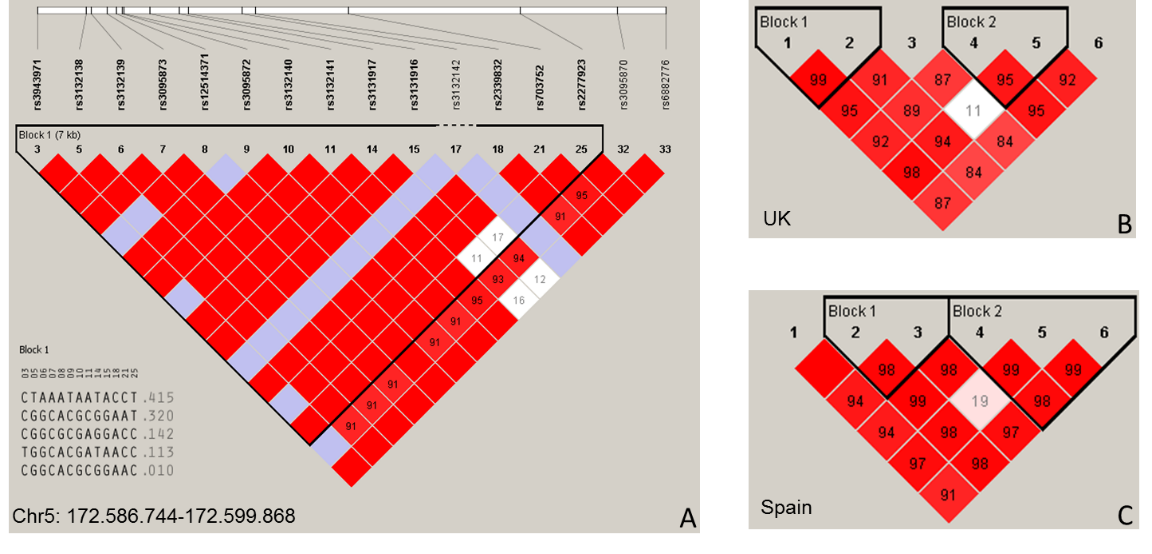


**Supplementary Figure 1. Linkage disequilibrium plots showing r^2^ values**. A. LD along the 13Kb of NKX2-5 genomic locus using genotype data for the CEU cohort of the HapMap project, and the haplotype blocks physically occur in the European population. B. LD plot using the genotype data for the UK cohort. C. LD plot using the genotype data for the replication cohort.

**Supplementary Figure 2**


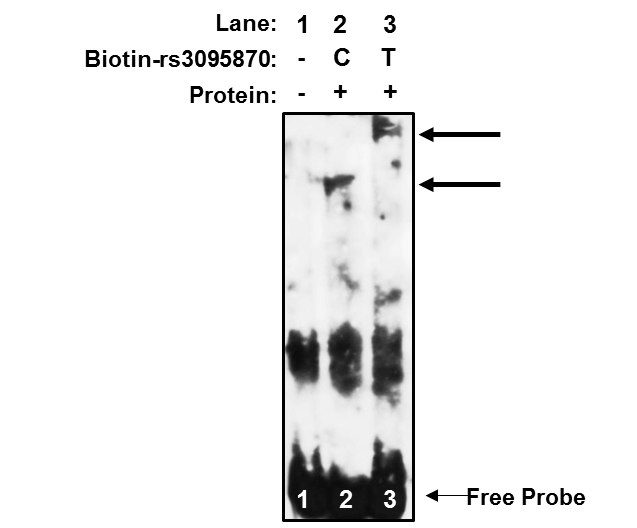


**Supplementary Figure 2**. **Electromobility shift assay.** EMSA in TGF-β treated ImHPASMC showing that the C and T alleles of rs3095870 have different binding affinities. The protein complexes formed are pointed with the arrows.

**Supplementary Figure 3**


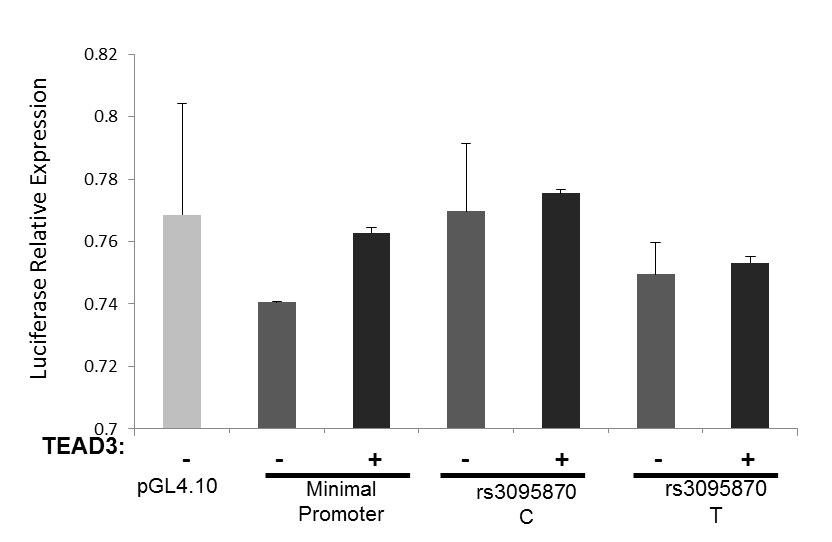


**Supplementary Figure 3.** **Luciferase reporter assays.** Co-transfection of TEAD3 together with the rs3095870 constructs did not change the luciferase expression, showing that TEAD3 alone is not able to enhance transcriptional activity.
